# Trade-Off and Synergy among Ecosystem Services in the Guanzhong-Tianshui Economic Region of China

**DOI:** 10.3390/ijerph121114094

**Published:** 2015-11-03

**Authors:** Keyu Qin, Jing Li, Xiaonan Yang

**Affiliations:** Tourism and Environment College, Shaanxi Normal University, Xi’an 710062, Shaanxi, China; E-Mails: qdqky924@126.com (K.Q.); yangxiaonan@snnu.edu.cn (X.Y.)

**Keywords:** Guanzhong-Tianshui economic region, ecosystem service, trade-off, synergy

## Abstract

Natural ecosystems provide society with important goods and services. With rapidly increasing populations and excessive utilization of natural resources, humans have been enhancing the production of some services at the expense of others. Although the need for certain trade-offs between conservation and development is urgent, having only a small number of efficient methods to assess such trade-offs has impeded progress. This study focuses on the evaluation of ecosystem services under different land use schemes. It reveals the spatial and temporal distributions of and changes in ecosystem services. Based on a correlation rate model and distribution mapping, the trade-offs and synergies of these ecosystem services can be found. Here, we also describe a new simple approach to quantify the relationships of every trade-off and synergy. The results show that all ecosystem services possess trade-offs and synergies in the study area. The trend of improving carbon sequestration and water interception indicate that these key ecosystem services have the strongest synergy. And the decrease in regional agricultural production and other services, except water yield, may be considered as trade-offs. The synergy between water yield and agricultural production was the most significant, while the trade-off between water interception and carbon sequestration was the most apparent, according to our interaction quantification model. The results of this study have implications for planning and monitoring the future management of natural capital and ecosystem services, and can be integrated into land use decision-making.

## 1. Introduction

Ecosystem services refer to the provisions or services that are produced (directly or indirectly) by an ecosystem. They can be utilized for human well-being actively or passively [1]. It has been widely accepted that human activities are the main factor in the transformation of the Earth’s surface [2,3]. Many researchers have suggested that there is a greater need to focus on the interactions among multiple services in order to produce more outcomes for human well-being [4,5]. To make better decisions, a systematic account of the relationships between ecosystem management and the generated ecosystem services is needed [6]. However, until now, the great majority of government or company policies has been concerned about only one factor at a time instead of taking full account of other [7,8,9,10]. Much work has been done to explore trade-offs and synergies in order to reveal the interactions that really occur among multiple ecosystem services. Less work has been done to quantify these interactions. The quantification of trade-off and synergy has strong implications for ecosystem management [11].

Ecosystem service trade-offs arise when the provision of one service is enhanced at the cost of reducing the provision of another service, and ecosystem service synergies arise when multiple services are enhanced simultaneously [12,13,14,15,16,17,18,19,20,21,22]. For example, the establishment of reserves or reducing the amount of water for agriculture would certainly cause a loss in grain production. Carbon sequestration, bird habitat provision and hay production are greater in wetlands at the cost of reducing grazing quality and plant diversity [23]. Enhancing the supply of certain ecosystem services, such as Soil and Water Conservation, and Carbon Sequestration and Oxygen Release, has led to the decline in many other ecosystem services, such as the production of grain or timber [20,24,25]. The most direct trade-off in floodplain services is food and fiber production (farmers) *versus* water quality regulation [26]. In contrast, one service, such as enhancing Carbon Sequestration and Oxygen Release, arises by increasing vegetation cover, which can lead to the enhancement of water conservation and the reduction of the wind effect. And improving nutrient retention by promoting vegetated riparian zones can leads to enhancements of wind protection, crop production, landscape beauty and water quality [27,28,29]. All of these consequences would benefit our society [27,30]. Research has proved that changes in land use may significantly affect ecosystem services and processes [22,31]. When decision-makers and scientists set out to explore possible land use, they need information about the ecological functions and how changes in the mix of land use would impact the trade-offs between production needs and other human needs. By finding out how the interactions work, we could maximize the values we desire by enhancing synergism or mitigating trade-offs [32]. Consequently, ascertaining the trade-offs and synergies among ecosystem services might improve the practices based on ecosystem management, and might help governments and companies to achieve their goals [33].

How to make full use of trade-offs and synergies among ecosystem services is vitally important to western China, which has a severe contradiction between its fragile ecosystem and the growing need of the increasing population for increased output from the land [34,35]. The Guanzhong-Tianshui Economic Region has a large population, as well as a series of conservation areas, such as the Loess Plateau Protected Area, and a part of the Grain for Green Project Conservation Area. Due to natural conditions and human activity in recent years, the Wei river flow, which is the biggest tributary of the Yellow River, has been polluted with urban domestic sewage, industrial waste, and run-off from agricultural pesticides and fertilizers. Hence, research into the ecosystem services of the economic region has realistic demands. Studies have shown that a change in land use models could greatly influence ecological systems [35]. A number of ecosystem conservation set-aside programs in China, such as the Grain for Green Program (changing sloping farmland into forest) and the Restoring Grazing to Grassland Project (changing sloping farmland into natural grassland), have testified to the possibility of improving ecosystem services by decreasing water surface runoff and soil erosion, and by increasing vegetation cover [36,37,38]. Using some models and remote sensing data, this study focuses on the evaluation of the supplies of ecosystem services under different land use schemes, and the synergies and trade-offs between them. This study has managed to use a simple method (by using the root mean square deviation) to not only reveal the interactions, but also to quantify all trade-offs and synergies.

## 2. Method

### 2.1. Study Area

**Figure 1:**
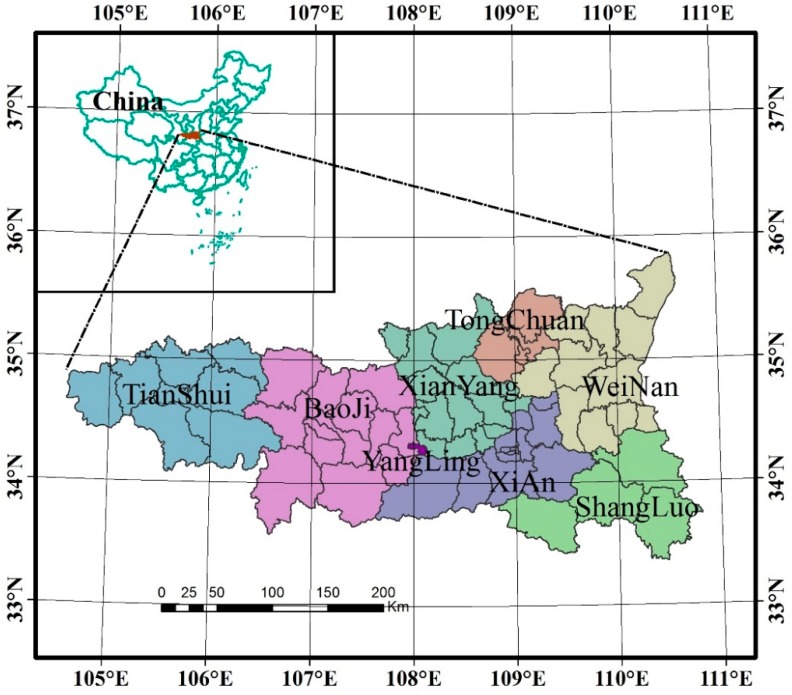
Administrative map of the study area.

The Guanzhong-Tianshui Economic Region (104°34′ E–110°48′ E 33°21′ N–35°51′ N) is comprised of the administrative regions of Xi’an, Tongchuan, Baoji, Xianyang, Weinan, Yangling and Shangluo (some prefectures and counties) of Shaanxi Province, as well as Tianshui of Gansu Province (Figure 1). It covers a total area of 79,800 square kilometers and has a total population of 28.42 million people, as of the end of 2010.

The Guanzhong-Tianshui Economic Region is located in the monsoon area of transition from semi-humid to semi-arid. It has a various topographic type, rich soil and land resources. It is an important strategic place that connects the east to the west of China, and the south to the north. Main land use types of the region are grain land, forest land, grassland, water area, urban land and unused land. The economic region boasts a favorable economic foundation in west China, advanced natural conditions, a profound human history and huge development potential.

### 2.2. Data Source

The data of land use were obtained from the cloud-free TM remote sensing images (2000, 2005 and 2010) that were downloaded from the Geospatial Data Cloud (http://www.gscloud.cn). The resolution of the images are 30 m × 30 m, and each image covers 295,583 square kilometers. The social and economic data were obtained from the STATISTICAL YEARBOOK OF GUANGZHONG-TIANSHUI, the STATISTICAL YEARBOOK OF SHANXI PROVINCE and the STATISTICAL YEARBOOK OF GANSU PROVINCE, *etc.* Data about the climates of the Guanzhong-Tianshui economic region were obtained from the meteorological department. The topographical information used in this study was derived from a Digital Elevation Model (DEM) with a resolution of 25 m × 25 m, which had been purchased from the National Geomatics Center of China. The soil data, including a soil type map (1:100,000), were obtained from the Soil Survey Office of Shaanxi Province. Watershed management information was added to improve the modeling accuracy. The watershed climatic features were simulated based on daily historical monitoring data from 33 weather stations from 1954 to 2010. The vegetation map of China was at a scale of 1:100,000. Details of the data sources are shown in Table 1.

**Table 1:** Data and data sources.

Required Data	Soil and Water Conservation	Water Interception	Carbon Sequestration	Agricultural Production	Water Yield	Data Source
Land-Use Map	▬	▬				Landsat TM
DEM	▬					Topographic Map of 1:50,000
Soil Type	▬					Agricultural Sector
Rainfall	▬				▬	Agricultural Sector
Temperature			▬			Agricultural Sector
Evaporation			▬		▬	Meteorological Department
Solar Radiation			▬			Meteorological Department
Administrative Map	▬	▬	▬			Civil Affairs
Grain Production				▬		Statistical Yearbook

▬ represents the source of study data.

### 2.3. Methods of Analysis

#### 2.3.1. Water Yield

The amount of water yield can be calculated based on the Budyko curve, which expresses the close relationship between factors of climate and the water cycle [39]. The water yield for each pixel on the landscape can be defined as follows:

(1) $$Y_{xj}=\left(1-\frac{ET_{xj}}{P_{x}}\right)\times P_{x}$$

where *Y_xj_* is the annual water yield for each pixel x on landscape j. *ET_x_* is the annual actual evapotranspiration on pixel x on landscape j, and *P_x_* is the annual precipitation on pixel x.

The monetary value of the water yield can be calculated by the engineering substitution method. The theory is based on the assumption that there is a water storage project that can store the same amount of water produced by the ecosystem. Then, the value of the water yield could be replaced by the amount of water stored by that project.

#### 2.3.2. Carbon Sequestration

The amount of carbon sequestration can be determined from the net primary productivity (NPP), which refers to the amount of organic material created by green plants per unit area in a given amount of time. We used the Carnegie–Ames–Stanford Approach (CASA) model to measure the NPP [40]. Specifically, the NPP is calculated using an expression such as that below:

(2) NPP(x,t)=APAR(x,t)×ε(x,t)

Here, APAR(x,t) denotes the amount of photosynthetically active radiation absorbed by element x in month t, while ε(x,t) is a factor that reflects the efficiency with which light energy is used to produce organic compounds in element x for month t  APAR(x,t) is calculated using the following expressions [40]:

(3) APAR(x,t)=SOL(x,t)×FPAR(x,t)×0.5

(4) $$FPAR\left(x,t\right)=\left(FPAR{\left(x,t\right)}_{NDVI}+FPAR{\left(x,t\right)}_{SR}\right)/2$$

(5) $$FPAR{\left(x,t\right)}_{NDVI}=\frac{\left(NDVI\left(x,t\right)-NDVI_{i,min}\right)\times \left(FPAR_{max}-FPAR_{min}\right)}{\left(NDVI_{i,max}-NDVI_{i,min}\right)}+FPAR_{min}$$

(6) $$FPAR{\left(x,t\right)}_{SR}=\frac{\left(SR\left(x,t\right)-SR_{i,min}\right)\times \left(FPAR_{max}-FPAR_{min}\right)}{\left(SR_{i,max}-SR_{i,min}\right)}+FPAR_{min}$$

(7) $$SR\left(x,t\right)=\frac{\left(1+NDVI\left(x,t\right)\right)}{\left(1-NDVI\left(x,t\right)\right)}$$

where SOL(x,t) denotes the solar bolometric radiation (MJ/m^2^). FPAR(x,t) is the fraction of the total incident PAR absorbed by the vegetation. The constant 0.5 reflects the proportion of the incident solar radiation that is photosynthetically active (*i.e.*, the proportion with a wavelength between 0.38 and 0.71 u m). $FPAR{\left(x,t\right)}_{NDVI}$ and $FPAR{\left(x,t\right)}_{SR}$ are factors for relating measured NDVI and Surface Reflectance (SR) values, respectively, to the APAR. $FPAR_{max}$ and $FPAR_{min}$ are independent of the cover type and take values of 0.95 and 0.001, respectively. $NDVI_{i,min}$ and $NDVI_{i,max}$ denote the minimum and maximum NDVI values observed for the i-th landscape type. Similarly, $SR_{i,max}$ and $SR_{i,min}$ are the maximum and minimum surface reflectance values for the i-th landscape type. NDVI(x,t) and SR(x,t) denote the normalized vegetation index and the surface reflectance for element x in month t.

It has been argued that the maximum possible light use efficiency under specific conditions is determined by the temperature and moisture, as shown below [41]:

(8) $$\varepsilon \left(x,t\right)=T_{\varepsilon 1}\left(x,t\right)\times T_{\varepsilon 2}\left(x,t\right)\times W_{\varepsilon }\left(x,t\right)\times \varepsilon_{max}$$

Here $T_{\varepsilon 1}\left(x,t\right)$, $T_{\varepsilon 2}\left(x,t\right)$ and $W_{\varepsilon }\left(x,t\right)$ are parameters describing the effects of low temperatures, high temperatures and moisture on the efficiency of light use. $\varepsilon_{max}$ is the maximum possible efficiency (g C·MJ^−1^) under ideal conditions. These parameters can be computed as described by [42]:

(9) $$T_{\varepsilon 1}\left(x,t\right)=0.8+0.02\times T_{opt}\left(x\right)-0.0005\times {\left[T_{opt}\left(x\right)\right]}^{2}$$

(10) $$T_{\varepsilon 2}\left(x,t\right)=\frac{1.184}{\left\{1+\exp\left[0.2\times \left(T_{opt}\left(x\right)-10-T\left(x,t\right)\right)\right]\right\}}\times \frac{1}{\left\{1+\exp\left[0.3\times (-T_{opt}\left(x\right)-10+T(x,t))\right]\right\}}$$

(11) $$W_{\varepsilon }\left(x,t\right)=0.5+0.5\times E\left(x,t\right)/E_{p}\left(x,t\right)$$

(12) $$E(x,t)=\frac{\left\{P\left(x,t\right)\times R_{n}\left(x,t\right)\times \left[{\left(P\left(x,t\right)\right)}^{2}+{\left(R_{n}\left(x,t\right)\right)}^{2}+P\left(x,t\right)\times R_{n}\left(x,t\right)\right]\right\}}{\left\{\left[P(x,t)+R_{n}(x,t)\right]\times \left[{\left(P\left(x,t\right)\right)}^{2}+{\left(R_{n}\left(x,t\right)\right)}^{2}\right]\right\}}$$

(13) $$R_{\text{n}}\left(x,t\right)={\left[E_{po}\left(x,t\right)\times P\left(x,t\right)\right]}^{0.5}\times \left\{0.369+0.589\times {\left[\frac{E_{po}\left(x,t\right)}{P\left(x,t\right)}\right]}^{0.5}\right\}$$

(14) $$E_{\text{p}}\left(x,t\right)=\left[E\left(x,t\right)+E_{po}\left(x,t\right)\right]/2$$

Here, $T_{opt}\left(x\right)$ is the average temperature for the month with the highest recorded NDVI for the study area. E(x,t) is the measured evapotranspiration for the region, P(x,t) is the precipitation (mm) that fell on element x in month t, $R_{n}\left(x,t\right)$ is the solar radiation incident on element x in month t, and $E_{po}\left(x,t\right)$ denotes the local latent evapotranspiration (mm). 

The amount value of carbon sequestration by an ecosystem can be determined from the net primary productivity: for every kilogram of dry matter produced, 1.63 kg of CO_2_ must have been fixed. The value of CO_2_ sequestration, which is approximately 260.90 RMB/t, can be calculated by the afforestation cost method [43].

#### 2.3.3. Water Interception

Water interception through land use is made up of three parts: canopy interception, litter containment and soil containment [44]. The goods model is as follows:

(15) $$Q_{t}=W_{1}+W_{2}+W_{3}$$

where *W_1_*, *W_2_*, *W_3_*, refer to amount of canopy, litter and soil respectively. *Q_t_* refers to the total amount of water interception.

The valuation method of land use ecosystem services for water interception can be described by the following equation:

(16) $$E_{w}=Q\cdot k$$

where $E_{w}$ is the value of water interception (in RMB unit), Q is the total amount of water interception. k is the unit water interception cost, and can be estimated by the engineering substitution method. In China, the cost of constructing a reservoir is 0.67 RMB per 1 m^3^. So here, k = 0.67 RMB/m^3^. 

#### 2.3.4. Soil Conservation

The Universal Soil Loss Equation (USLE) is an erosion estimation model to assess the soil losses that would generally result from splash, sheet and rill erosion [45]. The USLE was applied in GIS software to determine the average annual soil loss and its distribution in the study area. The amount of soil conserved by land use ecosystems can be estimated by the difference between potential and real soil erosion [6,46]:

(17) $$A_{c}=A_{p}-A_{r}$$

(18) $$A_{p}=R\times K\times L\times S$$

(19) $$A_{r}=R\times K\times L\times S\times C\times P_{s}$$

where $A_{c}$ is the amount of soil conservation (t/hm^2^·a); $A_{p}$ is the amount of potential soil erosion (t/hm^2^·a); $A_{r}$ is the amount of real soil erosion (t/hm^2^·a); *R* is erosion index by rainfall (Ft·T·In/A·h); *K* is soil erosion factor; L is length of slope, *S* is slope; *C* is vegetation cover factor; and *P_s_* is soil conservation measure factor.

Using market prices, opportunity costs and alternative projects, the value of the soil conservation of land-use is calculated for its role in conserving soil fertility, reducing land abandonment and reducing sediment accumulation.

According to the law of mud and sand motion in the major valleys in China, 24% of mud and sand accumulates in reservoirs, rivers and lakes. The value of reducing sediment accumulation by land-use ecosystem can be calculated by water storage costs. The model is as follows:

(20) $$E_{n}=A_{c}\times Rs\times Cs$$

where $E_{n}$ is the value of reducing sediment accumulation (in RMB unit), *A_c_* is the amount of soil conservation (ton), $C_{s}$ is reservoir construction cost (RMB/m^3^), and $R_{s}$ is soil bulk density (t/m^3^).

Land area abandoned by soil erosion can be calculated by the amount of soil conservation and the average thickness of surface soil (0.6 m). Using opportunity costs, the annual loss in value caused by land area abandonment can be calculated by:

(21) $$E_{S}=A_{C}\times B÷P÷0.6$$

where $E_{s}$ is the annual loss value caused by land area abandoned (in RMB unit), $A_{c}$ is the amount of soil conservation (ton), *B* is the annual income from forestry (RMB/hm^2^), and *P* is soil bulk density (t/m^3^).

Soil erosion causes a great deal of loss of nutrient matter, especially *K*, *P* and *N*. The content of *K*, *P* and *N* varies greatly among different soil types. Based on GIS, the mean values of *K*, *P* and *N* for each of the various land use ecosystems can be calculated. The value model of soil fertility conserved by land use ecosystems is as follows:

(22) $$E_{f}=\sum A_{C}C_{i}P_{i}$$

where $E_{f}$ is the value of soil fertility conservation (in RMB unit); $A_{c}$ is the amount of soil conservation; $C_{i}$ is the pure content of *N*, *P*, and *K*; and $P_{i}$ is the price of *N*, *P* and *K* (in RMB unit).

#### 2.3.5. Agricultural Production

We calculated the average yield, price and cost of the agricultural production of each commodity according to agricultural censuses taken in 2000, 2005 and 2010, and from state government Gross Margin Handbooks (county scale).

#### 2.3.6. Finding Out and Quantification of Trade-Offs and Synergies

With the ecological service of each grid as the data, using R software, a correlation analysis was carried out on the five kinds of ecological services, resulting in the correlations among water yield, carbon sequestration, water interception, soil conservation and agricultural production. All of these services present trade-offs and synergies. In fact, agricultural production was only carried out in land farmed for grain, so we set the average agricultural production value only to grid cells marked as grain land, and set 0 to other land use types. In order to ensure the accuracy of quantification, we analyzed the mean value of each ecosystem service in the six types of land use each year, then normalized the data to find the spatial differences of the relationships among ecological services in the land-use scale from 2000 to 2010.

**Figure 2:**
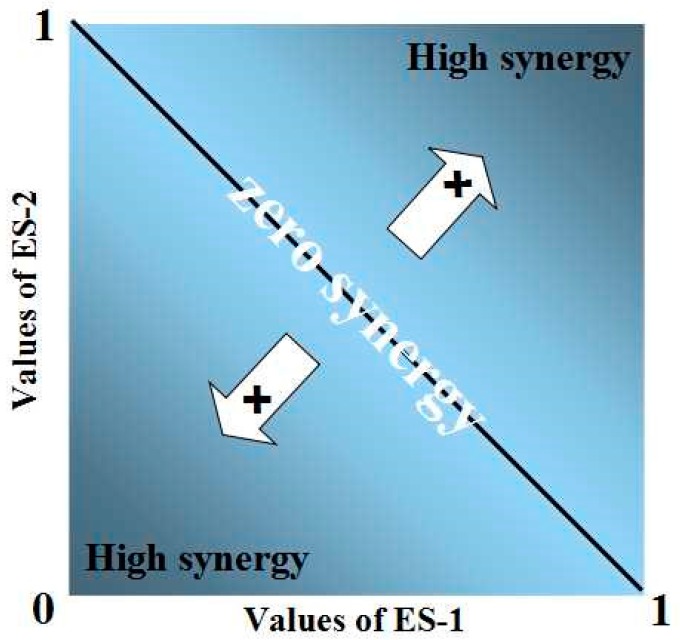
Illustration of overall synergy between two ecosystem services. Illustration of overall synergy between two ecosystem services. The overall Synergy is calculated as the RMSE of the individual benefits and increases with the distances from the reverse 1:1 line.

**Figure 3:**
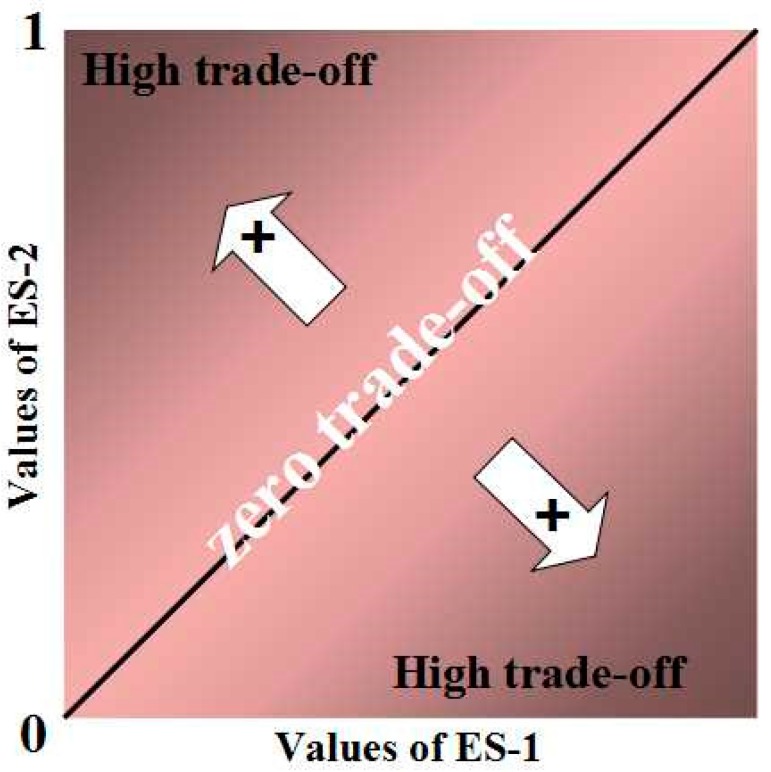
Illustration of overall trade-off between two ecosystem services. Illustration of overall trade-off between two ecosystem services. The overall Trade-off is calculated as the RMSE of the individual benefits and increases with distances from the 1:1 line.

In our study, we use an empirical model to quantify trade-offs and synergies among ecosystem services. Also, we have developed a new framework based on the existing empirical model [47]. In the new framework, we can quantify not only trade-offs, but also synergy. The magnitude of values of ecosystem services A is calculated as:

(23) $$Ma=\frac{Va-Va\min}{Va\max-Va\min}$$

where Va is the value of ecosystem service A, Vamax and Vamin are the maximum and minimum values of ecosystem A. Individual values range from 0 to 1. The values of each pixel are entered into the axes. The numbers on the x-axis stand for the values of ecosystem service A, while those on the y-axis stand for service B. The root mean square deviation (RMSD) of the two sets of values is calculated. RMSD means the distance between the actual points and the diagonal line (synergy: x + y −1 = 0 (Figure 2); trade-off: x − y = 0 (Figure 3)).The overall trade-offs and synergies for multiple ecosystem services can be estimated by taking the mean of the individual benefits.

## 3. Results

### 3.1. Temporal and Spatial Distribution Characteristics of Ecosystem Services

**Figure 4:**
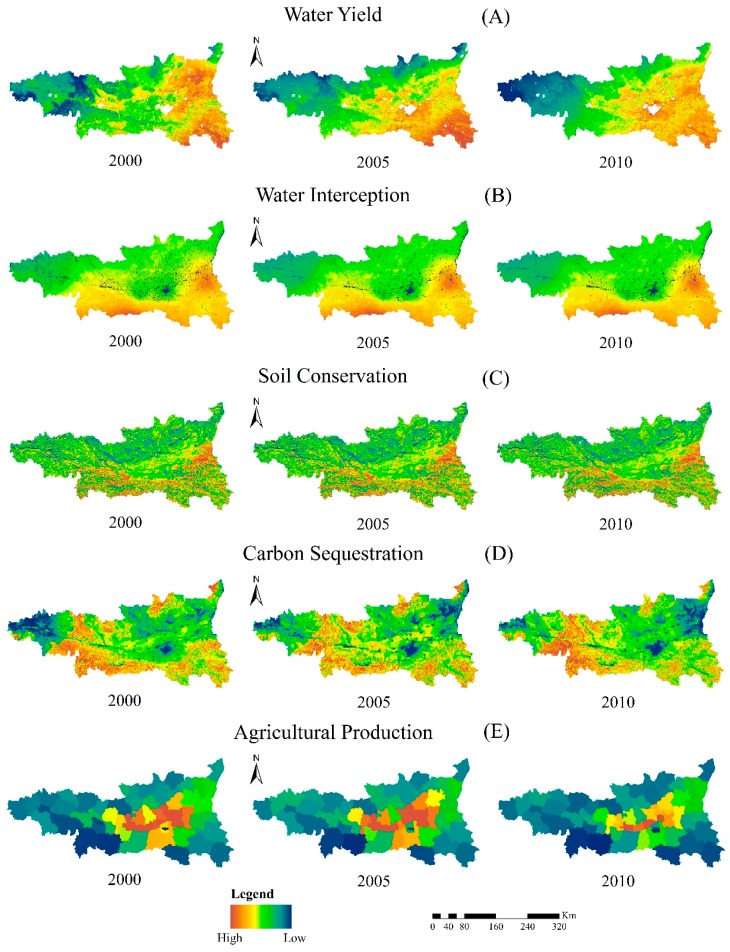
Maps of ecosystem services in the Guanzhong-Tianshui Economic Region.

The value of each ecosystem service from year 2000 to 2010 is shown in Figure 4. The spatial distributions of water yield, water interception and soil conservation are similar. The values of those ecosystem services increase approximately from west to east, and from north to south. The distributions of carbon sequestration and agricultural production on spatial scales are quite different from those above. 

Values of carbon sequestration in the mid-western and southern locations are higher than the remaining areas within the study area. For agricultural production, the values in the middle of the region are higher than in the perimeter region. The values of carbon sequestration and soil conservation have grown much over time. The growth rate of carbon sequestration was 26.9% from 2000 to 2010, and of soil conservation was 149%. Changes in these two ecosystem services are also different in space. Carbon sequestration increases from east to west, while soil conservation increases from north to south. On the other hand, changes in water yield and water interception are small. Only a few increases happen to these two ecosystem services, but the amount of the whole values does not change much. For agricultural production, the whole values in 2010 were 1.8 times more than the values in 2000. The changes in Xi’an were much higher than those of other places within the study area.

### 3.2. Interactions among Ecosystem Services

**Figure 5:**
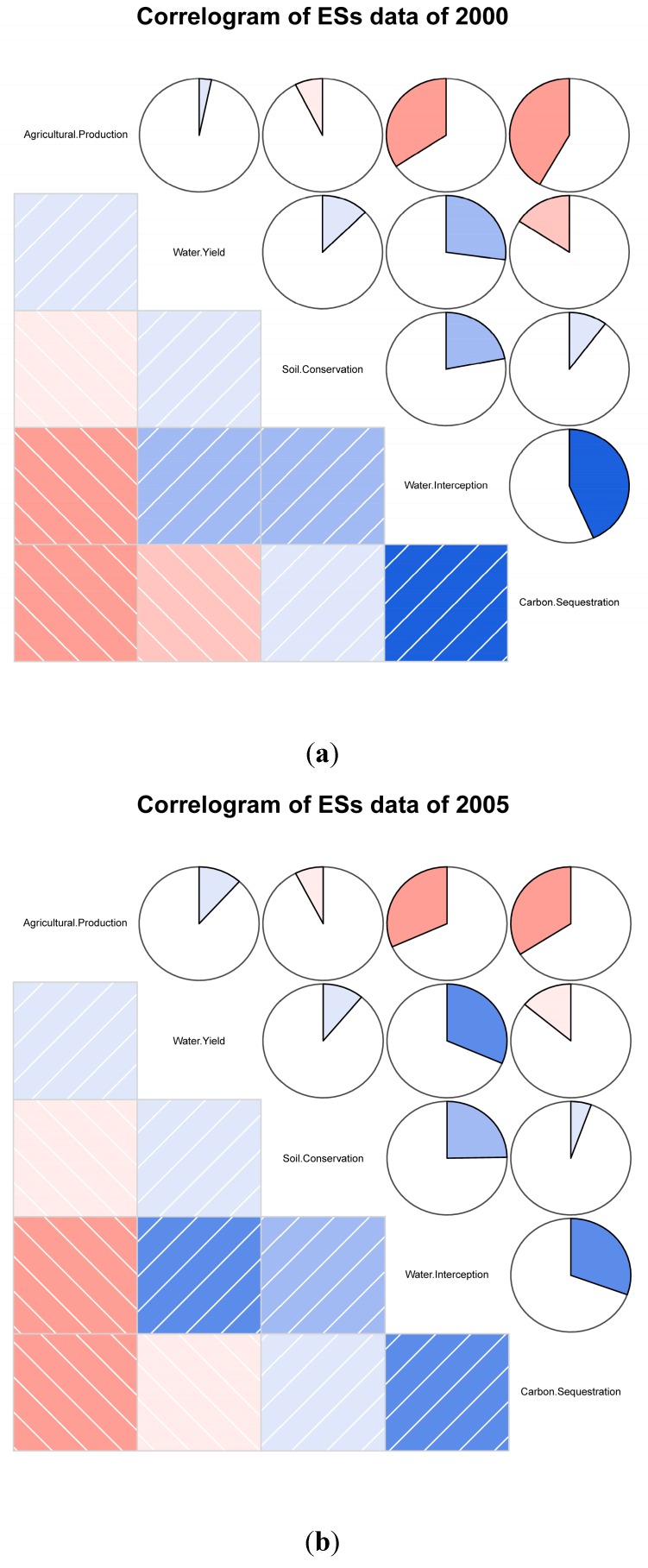

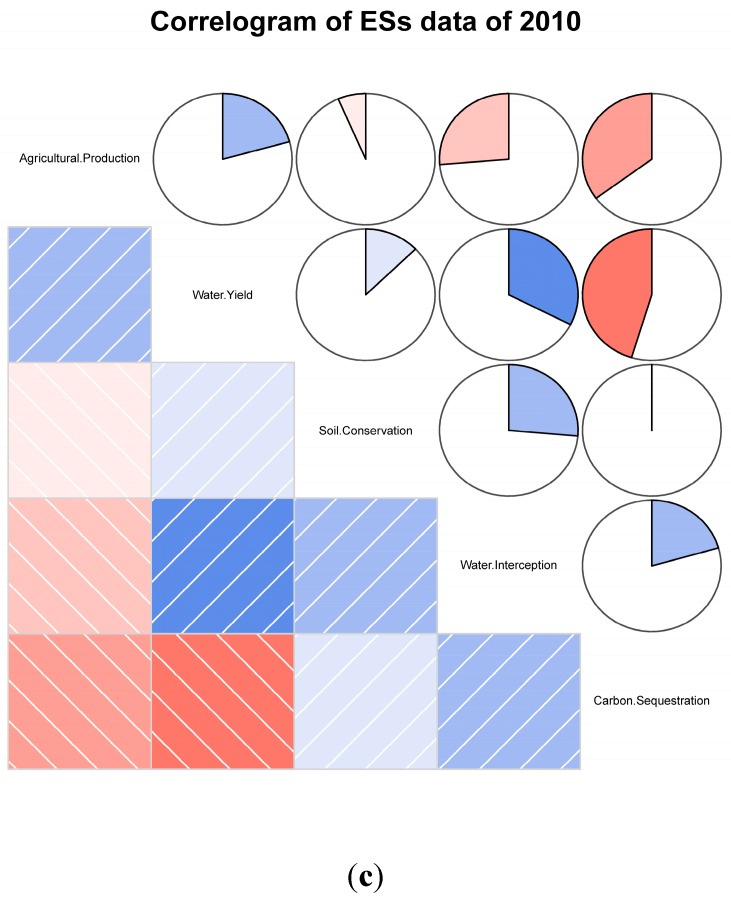
Correlograms of ecosystem services. In the above plot, the directions of slashes in the lower panel divide relationships into two categories, positive and negative. Also, blue denotes positive relationships while pink denotes negative relationships. The darker the colors and the bigger the painted areas are, the stronger the relationships between data. Color covering the whole right pie means the correlation coefficient equals to 1. And an empty pie means 0 correlation coefficient. (**a**) for year 2000, (**b**) for year 2005 and (**c**) for year 2010.

Our results reveal strong trade-offs and synergies among ecosystem services at the grid scale (Figure 5). The strongest trade-offs exist between agricultural production and carbon sequestration in the years 2000 and 2005. In 2010, strong trade-offs existed between water yield and carbon sequestration. The strongest synergies existed between water yield and water interception in 2005 and 2010, and between water interception and carbon sequestration in 2000. Our study shows that trade-offs exist between agricultural production and almost all other ecosystem services, with the exception of water yield. All of the ecosystem services in our study have trade-offs or synergies among them.

**Figure 6:**
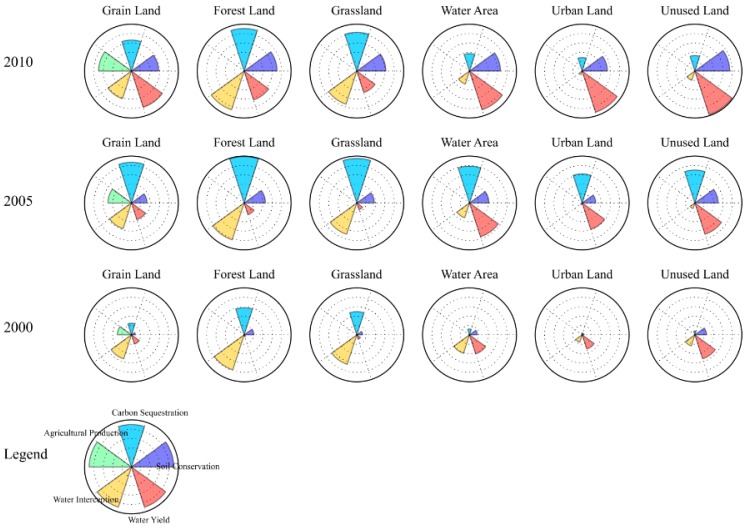
Flower diagrams. Reveals the normalized value of each ecosystem service through petal length, each flower represents one land-use type in a certain year.

By using flower diagrams, we also find out that on the temporal scale, almost all values have an increasing trend, mainly because prices have appreciated much in China from 2000 to 2010. On the spatial scale, water interception and carbon sequestration have a clear synergy among the land use types in each year. Water yield and carbon sequestration have trade-offs among land use types (Figure 6).

### 3.3. Quantification of Trade-Offs and Synergies

In this study, we used statistical analysis to improve the accuracy of our quantification. We have identified trade-offs and synergies among the values of all five kinds of ecosystem services. And by using the model of trade-off quantification and synergy quantification respectively, we calculate the indexes of both trade-offs and synergies for each ecosystem service (Table 2, in 2000) Water yield and agricultural production have the highest levels of synergy of all the years (0.30 in years 2005 and 2010, 0.27 in 2000), while water interception and agricultural production have the highest level of trade-offs (0.50 in 2010, 0.44 in 2005, and 0.45 in 2000). Soil conservation and carbon sequestration have the lowest levels of synergy in 2005 and 2010 (synergy index is 0.13). In 2000, water interception and soil conservation were the lowest (0.15). On the other hand, water yield and carbon sequestration had the lowest level of trade-offs (0.16 in years 2000 and 2005, 0.19 in 2010).

## 4. Discussion

Our ecosystem services model focuses on water yield, carbon sequestration, water interception and soil conservation. Along with remote-sensing (RS) techniques and a geographic information system (GIS), a number of RS models, which were well developed for studying the NPP and carbon cycle on global and regional scales, were used to calculate vegetation NPP [48]. The CASA model has been widely used in China [48,49,50]. The Budyko curve for calculating the water yield simplifies the convergence process, which helped us to simulate the distribution of regional water yield [51].

The USLE model for evaluating soil conservation is popular for applications in the grid environment with GIS, because it allow us to analyze soil losses in much more detail since the process has a spatially distributed character. GIS-based plain management using USLE has the potential to alleviate soil erosion in the region and can play significant roles in generating parameters from remote areas for watershed management. Afforestation method has been used when calculating the value of carbon sequestration in our study. But while 1.63 kg of CO_2_ were sequestrated, 1.2 kg of oxygen for every kilogram of net primary production also released. Further study should consider the value of the released oxygen.

Growing evidence shows that management options that are beneficial for one ecosystem service might lead to trade-offs and synergies that increase or decrease the values of other ecosystem services. Frameworks and structures used to evaluate the size of trade-offs and synergy are still not applicable in many cases, and are often too complicated for decision-makers [52,53,54]. Few approachable common methods have been explored to quantify those interactions until now. In our study, we make a simple step to quantify the index of trade-off and synergy by calculating the RMSE of the distance between each value point and the 1–1 line. Future works should test much more complicated ecosystem services among multiple ecosystem services.

**Table 2:** Index of synergies and trade-offs among ecosystem services (green blanks for synergies and orange for trade-off).

	Water Yield	Water Interception	Soil Conservation	Carbon Sequestration	Agricultural Production
**2010**					
Water Yield	-				
Water Interception	0.29	-			
Soil Conservation	0.18	0.15	-		
Carbon Sequestration	0.19	0.27	0.13	-	
Agricultural Production	0.30	0.50	0.24	0.40	-
**2005**					
Water Yield	-				
Water Interception	0.23	-			
Soil Conservation	0.20	0.15	-		
Carbon Sequestration	0.16	0.25	0.13	-	
Agricultural Production	0.30	0.44	0.21	0.33	-
**2000**					
Water Yield	-				
Water Interception	0.25	-			
Soil Conservation	0.17	0.15	-		
Carbon Sequestration	0.16	0.26	0.16	-	
Agricultural Production	0.27	0.45	0.21	0.34	-

Ecosystem services are provided to agriculture at varying scales, and this can influence a user’s incentives for protecting the ecosystem service [55]. The maximum carbon sequestration has considerable impact on water interception and soil conservation, which can cause losses in agricultural production and water yield. All of these services present trade-offs and synergies. The users often retain existing practices after it is profitable to change land use [56]. When positive and public benefits can be achieved, policymakers must use some methods to increase the desired practice. Relative variances in water yield, carbon sequestration, water interception, soil conservation and agricultural production were found in this study. We modeled the spatial distributions of these processes by integrating a range of economic and water data that change in their spatial scale and detail, thereby affecting the relative variances in costs and benefits. However, it is the most significant limitation of this study that the model parameters may be uncertainty and variety, such as agricultural productivity and land use change adoption. The assumptions made of water interception in the model are extensive, in particular the linear relationship between water-soil-biomass regimes. This adds some modeling uncertainties that may affect the results presented here.

Some researchers have studied food-carbon trade-offs [56,57], interactions of the supply of carbon sequestration and biodiversity [58], carbon and water trade-offs [12,59,60,61], and trade-offs and synergies among food provision, biodiversity conservation, carbon sequestration and protection against natural hazards [9,11,62,63,64]. But few of them trying to quantify the interactions between ecosystem services. Tradeoffs were widely found between provisioning services and regulating services as our studies has proved. Compared to previous studies, this paper demonstrates a modeling approach that includes feedback loops and interactions among the ecosystem services in China, so that the trade-offs and synergies among these services become more explicit. However, the ecosystem services in the reforestation lists can be of at least 20 kinds [65], such as water quality and dry-land salinization. Future assessments should quantify the co-benefits and trade-offs for a broader range of ecosystem services among all land uses. 

## 5. Conclusions

Our results reveal strong trade-offs and synergies among ecosystem services by using correlation analysis. And by using the model of trade-off quantification and synergy quantification respectively, we calculate the indexes of both trade-offs and synergies for each ecosystem service. Water yield and agricultural production have the highest levels of synergy of all the years, while water interception and agricultural production have the highest level of trade-offs. Soil conservation and carbon sequestration have the lowest levels of synergy in 2005 and 2010. In 2000, water interception and soil conservation were the lowest. On the other hand, water yield and carbon sequestration had the lowest level of trade-offs. These results can provide the basis for government and society to formulate better policies that could move us towards “win-win” scenarios for ecological environments and economic benefits.

## References

[1] Fisher B., Turner R.K., Morling P. (2009). Defining and classifying ecosystem services for decision making. Ecol. Econ..

[2] Vitousek P.M., Mooney H.A., Lubchenco J., Melillo J.M. (1997). Human domination of Earth’s ecosystems. Science.

[3] Foley J.A., Costa M.H., Delire C., Ramankutty N., Snyder P. (2003). Green surprise? How terrestrial ecosystems could affect earth’s climate. Front. Ecol. Environ..

[4] Daily G.C., Polasky S., Goldstein J., Kareiva P.M., Mooney H.A., Pejchar L., Ricketts T.H., Salzman J., Shallenberger R. (2009). Ecosystem services in decision making: Time to deliver. Front. Ecol. Environ..

[5] Kareiva P., Watts S., McDonald R., Boucher T. (2007). Domesticated nature: Shaping landscapes and ecosystems for human welfare. Science.

[6] Bai Y., Zheng H., Ouyang Z., Zhuang C., Jiang B. (2013). Modeling hydrological ecosystem services and tradeoffs: A case study in Baiyangdian watershed, China. Environ. Earth Sci..

[7] Aillery M., Shoemaker R., Caswell M. (2001). Agriculture and ecosystem restoration in South Florida: Assessing trade-offs from water-retention development in the Everglades agricultural area. Am. J. Agric. Econ..

[8] Backéus S., Wikström P., Lämås T. (2005). A model for regional analysis of carbon sequestration and timber production. For. Ecol. Manag..

[9] Phelps J., Friess D., Webb E. (2012). Win-win REDD+ approaches belie carbon-biodiversity trade-offs. Biol. Conserv..

[10] Tallis H., Polasky S. (2011). Assessing Multiple Ecosystem Services: An integrated tool for the real world. Natural Capital. Theory and Practice of Mapping Ecosystem Services.

[11] Kragt M.E., Robertson M.J. (2014). Quantifying ecosystem services trade-offs from agricultural practices. Ecol. Econ..

[12] Dymond J.R., Ausseil A.-G.E., Ekanayake J.C., Kirschbaum M.U. (2012). Tradeoffs between soil, water, and carbon—A national scale analysis from New Zealand. J. Environ. Manag..

[13] Goldstein J.H., Caldarone G., Duarte T.K., Ennaanay D., Hannahs N., Mendoza G., Polasky S., Wolny S., Daily G.C. (2012). Integrating ecosystem-service tradeoffs into land-use decisions. Proc. Natl. Acad. Sci. USA.

[14] Hicks C.C. (2011). How do we value our reefs? Risks and tradeoffs across scales in “biomass-based” economies. Coast. Manag..

[15] Hicks C.C., Graham N.A., Cinner J.E. (2013). Synergies and tradeoffs in how managers, scientists, and fishers value coral reef ecosystem services. Glob. Environ. Chang..

[16] Jindal R., Kerr J.M., Ferraro P.J., Swallow B.M. (2013). Social dimensions of procurement auctions for environmental service contracts: Evaluating tradeoffs between cost-effectiveness and participation by the poor in rural Tanzania. Land Use Policy.

[17] Lester S.E., Costello C., Halpern B.S., Gaines S.D., White C., Barth J.A. (2013). Evaluating tradeoffs among ecosystem services to inform marine spatial planning. Mar. Policy.

[18] Meehan T.D., Gratton C., Diehl E., Hunt N.D., Mooney D.F., Ventura S.J., Barham B.L., Jackson R.D. (2013). Ecosystem-service tradeoffs associated with switching from annual to perennial energy crops in riparian zones of the US Midwest. PLoS ONE.

[19] Raudsepp-Hearne C., Peterson G.D., Bennett E. (2010). Ecosystem service bundles for analyzing tradeoffs in diverse landscapes. Proc. Natl. Acad. Sci. USA.

[20] Rodríguez J.P., Beard T.D., Bennett E.M., Cumming G.S., Cork S.J., Agard J., Dobson A.P., Peterson G.D. Trade-offs across space, time, and ecosystem services. http://www.ecologyandsociety.org/vol11/iss1/art28/.

[21] Swallow B.M., Sang J.K., Nyabenge M., Bundotich D.K., Duraiappah A.K., Yatich T.B. (2009). Tradeoffs, synergies and traps among ecosystem services in the Lake Victoria basin of East Africa. Environ. Sci. Policy.

[22] Viglizzo E.F., Frank F.C. (2006). Land-use options for Del Plata Basin in South America: Tradeoffs analysis based on ecosystem service provision. Ecol. Econ..

[23] Acreman M., Harding R., Lloyd C., McNamara N., Mountford J., Mould D., Purse B., Heard M., Stratford C., Dury S. (2011). Trade-off in ecosystem services of the Somerset Levels and Moors wetlands. Hydrol. Sci. J..

[24] Millennium Ecosystem Assessment (2001). Millennium Ecosystem Assessment.

[25] Bennett E.M., Balvanera P. (2007). The future of production systems in a globalized world. Front. Ecol. Environ..

[26] Butler J.R., Wong G.Y., Metcalfe D.J., Honzák M., Pert P.L., Rao N., van Grieken M.E., Lawson T., Bruce C., Kroon F.J. (2013). An analysis of trade-offs between multiple ecosystem services and stakeholders linked to land use and water quality management in the Great Barrier Reef, Australia. Agric. Ecosyst. Environ..

[27] Tallis H., Kareiva P., Marvier M., Chang A. (2008). An ecosystem services framework to support both practical conservation and economic development. Proc. Natl. Acad. Sci. USA.

[28] Bennett E.M., Peterson G.D., Gordon L.J. (2009). Understanding relationships among multiple ecosystem services. Ecol. Lett..

[29] Carpenter S.R., Mooney H.A., Agard J., Capistrano D., DeFries R.S., Díaz S., Dietz T., Duraiappah A.K., Oteng-Yeboah A., Pereira H.M. (2009). Science for managing ecosystem services: Beyond the Millennium Ecosystem Assessment. Proc. Natl. Acad. Sci. USA.

[30] Boody G., Vondracek B., Andow D.A., Krinke M., Westra J., Zimmerman J., Welle P. (2005). Multifunctional agriculture in the United States. BioScience.

[31] López E., Bocco G., Mendoza M., Duhau E. (2001). Predicting land-cover and land-use change in the urban fringe: A case in Morelia city, Mexico. Landsc. Urban Plan..

[32] White C., Halpern B.S., Kappel C.V. (2012). Ecosystem service tradeoff analysis reveals the value of marine spatial planning for multiple ocean uses. Proc. Natl. Acad. Sci. USA.

[33] Carreño L., Frank F., Viglizzo E. (2012). Tradeoffs between economic and ecosystem services in Argentina during 50 years of land-use change. Agric. Ecosyst. Environ..

[34] Cao S., Li C., Cao S., Peng Z., Yang L. (2013). Change in ecosystem service value arising from land consolidation planning in Anhui Province. Asian Agric. Res..

[35] Jia X., Fu B., Feng X., Hou G., Liu Y., Wang X. (2014). The tradeoff and synergy between ecosystem services in the Grain-for-Green areas in Northern Shaanxi, China. Ecol. Indic..

[36] Li D., Bo F., Tao J. (2006). Achievements in and strategies for Grain to Green Program in Hunan Province. Hun. For. Sci. Technol..

[37] Long H., Heilig G., Wang J., Li X., Luo M., Wu X., Zhang M. (2006). Land use and soil erosion in the upper reaches of the Yangtze River: Some socio-economic considerations on China’s Grain-for-Green Programme. Land Degrad. Dev..

[38] Xu J., Yin R., Li Z., Liu C. (2006). China’s ecological rehabilitation: Unprecedented efforts, dramatic impacts, and requisite policies. Ecol. Econ..

[39] Budyko M.I. (1971). Climate and Life.

[40] Yuan J., Niu Z., Wang C. (2006). Vegetation NPP distribution based on MODIS data and CASA model—A case study of northern Hebei Province. Chin. Geograph. Sci..

[41] Potter C.S., Randerson J.T., Field C.B., Matson P.A., Vitousek P.M., Mooney H.A., Klooster S.A. (1993). Terrestrial ecosystem production: A process model based on global satellite and surface data. Glob. Biogeochem. Cycles.

[42] Wenquan Z., Yaozhong P., Jinshui Z. (2007). Estimation of net primary productivity of Chinese terrestrial vegetation based on remote sensing. Chin. J. Plant Ecol..

[43] Zhiyuan R., Yanfang Z., Jing L. (2003). The value of vegetation ecosystem services: A case of Qinling-Daba Mountains. J. Geogr. Sci..

[44] Jing L., Zhiyuan R. (2011). Variations in ecosystem service value in response to land use changes in the Loess Plateau in northern Shaanxi Province, China. Int. J. Environ. Res..

[45] Bagherzadeh A. (2014). Estimation of soil losses by USLE model using GIS at Mashhad plain, Northeast of Iran. Arab. J. Geosci..

[46] Su C., Fu B., Wei Y., Lü Y., Liu G., Wang D., Mao K., Feng X. (2012). Ecosystem management based on ecosystem services and human activities: A case study in the Yanhe watershed. Sustain. Sci..

[47] Bradford J.B., D’Amato A.W. (2011). Recognizing trade-offs in multi-objective land management. Front. Ecol. Environ..

[48] Gao Q., Li Y., Wan Y., Qin X., Jiangcun W., Liu Y. (2009). Dynamics of alpine grassland NPP and its response to climate change in Northern Tibet. Clim. Chang..

[49] Fang J., Song Y., Liu H. (2002). Vegetation-climate relationship and its application in the division of vegetation zone in China. Acta Bot. Sin..

[50] Piao S., Fang J., He J. (2006). Variations in vegetation net primary production in the Qinghai-Xizang Plateau, China, from 1982 to 1999. Clim. Chang..

[51] Canqiang Z., Wenhua L., Biao Z., Moucheng L. (2012). Water yield of Xitiaoxi River Basin based on INVEST modeling. J. Resour. Ecol..

[52] Alvarez M., Field D.B. (2009). Tradeoffs among ecosystem benefits: An analysis framework in the evaluation of forest management alternatives. J. For..

[53] Matthews K., Buchan K., Sibbald A., Craw S. (2006). Combining deliberative and computer-based methods for multi-objective land-use planning. Agric. Syst..

[54] Spies T.A., Johnson K.N., Burnett K.M., Ohmann J.L., McComb B.C., Reeves G.H., Bettinger P., Kline J.D., Garber-Yonts B. (2007). Cumulative ecological and socioeconomic effects of forest policies in coastal Oregon. Ecol. Appl..

[55] Power A.G. (2010). Ecosystem services and agriculture: Tradeoffs and synergies. Philos. Trans. R. Soc. B.

[56] Paterson S., Bryan B.A. (2012). Food-carbon trade-offs between agriculture and reforestation land uses under alternate market-based policies. Ecol. Soc..

[57] West P.C., Gibbs H.K., Monfreda C., Wagner J., Barford C.C., Carpenter S.R., Foley J.A. (2010). Trading carbon for food: Global comparison of carbon stocks *vs.* crop yields on agricultural land. Proc. Natl. Acad. Sci. USA.

[58] Bryan B., Nolan M., Harwood T., Connor J., Navarro-Garcia J., King D., Summers D., Newth D., Cai Y., Grigg N. (2014). Supply of carbon sequestration and biodiversity services from Australia’s agricultural land under global change. Glob. Environ. Chang..

[59] Chisholm R.A. (2010). Trade-offs between ecosystem services: Water and carbon in a biodiversity hotspot. Ecol. Econ..

[60] Jackson R.B., Jobbágy E.G., Avissar R., Roy S.B., Barrett D.J., Cook C.W., Farley K.A., le Maitre D.C., McCarl B.A., Murray B.C. (2005). Trading water for carbon with biological carbon sequestration. Science.

[61] Schrobback P., Adamson D., Quiggin J. (2011). Turning water into carbon: Carbon sequestration and water flow in the Murray-Darling Basin. Environ. Resour. Econ..

[62] Briner S., Huber R., Bebi P., Elkin C., Schmatz D.R., Grêt-Regamey A. (2013). Trade-offs between ecosystem services in a mountain region. Ecol. Soc..

[63] Hall J.M., van Holt T., Daniels A.E., Balthazar V., Lambin E.F. (2012). Trade-offs between tree cover, carbon storage and floristic biodiversity in reforesting landscapes. Landsc. Ecol..

[64] Schindler S., Sebesvari Z., Damm C., Euller K., Mauerhofer V., Schneidergruber A., Biró M., Essl F., Kanka R., Lauwaars S.G. (2014). Multifunctionality of floodplain landscapes: Relating management options to ecosystem services. Landsc. Ecol..

[65] Daily G.C., Matson P.A. (2008). Ecosystem services: From theory to implementation. Proc. Natl. Acad. Sci. USA.

